# Unloading Characteristics of Sand-drift in Wind-shallow Areas along Railway and the Effect of Sand Removal by Force of Wind

**DOI:** 10.1038/srep41462

**Published:** 2017-01-25

**Authors:** Jian-jun Cheng, Guo-Wei Xin, Ling-yan Zhi, Fu-qiang Jiang

**Affiliations:** 1 College of Water Resources and Architectural Engineering, Shihezi University, Shihezi Xinjiang 832003, China; 2 Northwest Research Institute Co., Ltd. of China Railway Engineering Corporation, 365 Minzhu East Road, Lanzhou 730000, China

## Abstract

Wind-shield walls decrease the velocity of wind-drift sand flow in transit. This results in sand accumulating in the wind-shadow zone of both windshield wall and track line, causing severe sand sediment hazard. This study reveals the characteristics of sand accumulation and the laws of wind-blown sand removal in the wind-shadow areas of three different types of windshield walls, utilizing three-dimensional numerical simulations and wind tunnel experiments and on-site sand sediment tests. The results revealed the formation of apparent vortex and acceleration zones on the leeward side of solid windshield walls. For uniform openings, the vortex area moved back and narrowed. When bottom-opening windshield walls were adopted, the track-supporting layer at the step became a conflux acceleration zone, forming a low velocity vortex zone near the track line. At high wind speeds, windshield walls with bottom-openings achieved improved sand dredging. Considering hydrodynamic mechanisms, the flow field structure on the leeward side of different types of windshield structures is a result of convergence and diffusion of fluids caused by an obstacle. This convergence and diffusion effect of air fluid is more apparent at high wind velocities, but not obvious at low wind velocities.

China is one of the countries with the highest distribution of desert railways in the world, which are prone to problems, as evidenced by the sandstorm disaster of the Lanxin Railway II from Lanzhou to Xinjiang^1^. Construction of the Lanxin Railway II in Xinjiang started towards the end of 2009, and was completed and opened to public transport by the end of 2014. The Lanxin Railway II in Xinjiang has a total length of 1,776 km, crossing the four wind zones: “An Xi Feng Kou”, “Hundred Miles”, “Yandun”, and “Thirty Miles”^2^. The length of potential sandstorm disaster areas along the railway is as long as 462 km and the Lanxin Railway II is also the first high-speed railway bypassing sandstorm disaster areas in China^3^. The train speed is fast and the requirements for the operating environment are strict. Therefore, many different wind-proof constructions are utilized such as subgrade windshield walls, bridge windscreens, and wind-proof tunnels, as shown in Fig. 1.

The railway mileage of the Lanxin Railway II in Xinjiang in the “Yan Dun” wind zone is approximately 52 km. This wind zone is located in the desert and has characteristics of high wind speed, long wind season, strong seasonality, and fast wind blow. Such strong winds can easily influence trains at high-speed operation^4^. To resist the harmful effects of such strong winds on trains, the Lanxin Railway II adopts subgrade windshield walls as a main wind-proof project in the “Yan Dun” wind zone, and its engineering scale ranks first among high-speed railway construction projects in the world. Informed by the micro-topography on both sides of the railway, windshield walls with heights between 3.8 m to 4.3 m were constructed on the windward side of the wind zone. A variety of reinforced concrete windshield walls were designed based on different wind strengths, directions, frequencies, terrains, and railway conditions in different wind zones^5^.

The employment of windshield walls reduces the damage for train operation to a certain extent^6^. However, since the outside sand-shield system of windshield walls cannot intercept the entire sand-drift flow, a small part of the total sand-drift still remains unblocked and moves with the wind near the railway. Nevertheless, when windshield walls shield the railway from detrimental wind-caused effects (addressing the strong wind which can cause damage to the trains), unloading problems of sand-drift in the air will be introduced^7^,^8^. Therefore, the rebuilding of existing windshield walls and clearing of railway sand sediment by reducing most of the wind energy, thus controlling the sand, is still necessary and requires engineering practice and scientific experimental research.

Based on one-year of monitoring the wind speed in the “Yan Dun” wind zone, and according to the track characteristics of the Lanxin Railway II in the “Yan Dun” wind zone, a life-sized track model was established. The sand sediment characteristics of solid windshield walls, hanging windshield walls, and bottom-opening windshield walls were utilized as engineering background, and numerical simulation as well as wind tunnel experiment were carried out. The unloading characteristics of sand-drift in front of and behind openings in solid windshield walls were measured *in situ*. Since the inherent unpredictability of wind speed, it is necessary to investigate the effect of sand sediment removal from the track via openings. Consequently, sand removal effects were tested at different wind speed conditions. Through field measurements, numerical simulations, and wind tunnel experiments, this study reveals sand control characteristics of three types of windshield walls subjected to the influence of different wind forces. In addition, the windshield walls were optimized and modified to take both wind proofing and sand-prevention into account. This study therefore, provides a scientific basis for the design of wind-proof and sand-proof projects along the railway.

## Statistics of wind speed in the “Yan Dun” wind zone

**Figure 2:**
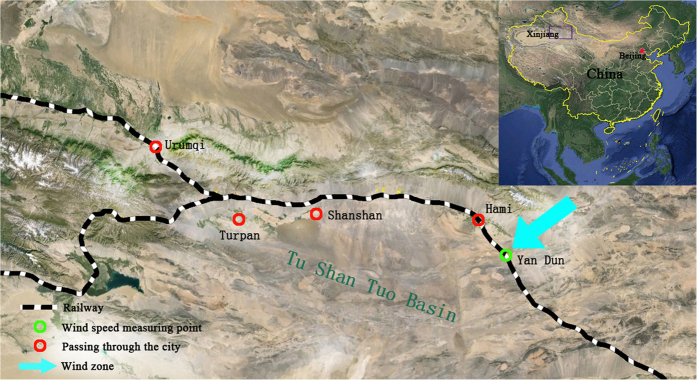
Location of the wind speed measuring point of the “Yan Dun” wind zone (The image is from 2016 Google Image Landsat/Copernicus Data SIO, NOAA, U.S. Navy, NGA,GEBCO).

Yan Dun is located in Daquan Town, near Hami City in the Xinjiang Uygur Autonomous Region of China, 1260 km from Lanzhou, and 650 km from Urumqi. The “Yan Dun” wind zone is located near Yan Dun to Jing Xia. It is one of the main wind zones along the Lanxin Railway. Therefore, wind speed data collected via measuring points in this area can reflect the overall situation of railways in the “Yan Dun” zone. Limited by experimental funds and field conditions, only one measuring point was set up in the “Yan Dun” wind zone, and the wind speed at a height of 10 m was monitored for one year to obtain wind speed data at the survey site (as shown in Fig. 2).

**Figure 3:**
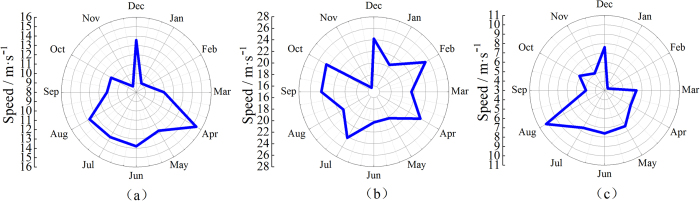
Rose diagrams of (**a**) average wind speed, (**b**) maximum wind speed, and (**c**) minimum wind speed from July 2011 to June 2012 measured at a height of 10 m at the “Yan Dun” measuring point.

Figure 3 shows the monthly average wind speeds, maximum wind speeds, and minimum wind speeds as rose diagrams ranging from July 2011 to June 2012 at a height of 10 m at the “Yan Dun” measuring point. Figure 3 reveals that the average annual wind speed in the “Yan Dun” wind zone was approximately 12 m·s^−1^, while the maximum wind speed was 25.3 m·s^−1^, and the minimum wind speed was 3.4 m·s^−1^.

## Results

### Results of the numerical analysis

#### Variation law of flow field

The wind speed near the surface has a significant effect on sand movement. Therefore, it is imperative to study and understand the change of near-surface wind velocities for the change of flow field and distribution of sand sediment around a windshield wall^9^. A wind speed of 15 m·s^−1^ was utilized as an example to simulate the change of flow field around the windshield wall. The velocity change of three windshield walls is shown in Fig. 3 (symmetry plane in xz direction).

**Figure 4:**
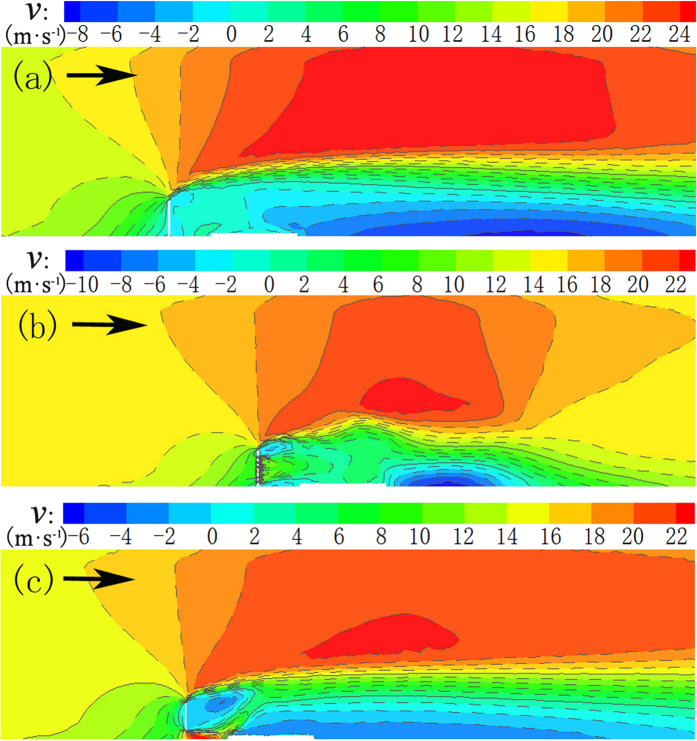
Distribution of flow fields in different types of windshield walls. (**a**) Solid type, (**b**) uniform-opening type, and (**c**) bottom-opening type.

Figure 4 reveals that the deceleration zone, acceleration zone, and vortex zone appear successively in the flow field, and the acceleration zone is located above the vortex zone. Due to different characteristics of the opening distribution of three types of windshield walls, there are different airflow phenomena on the leeward-side of windshield walls: the vortex and acceleration zones were formed on the leeward side of the solid windshield wall, and the resulting wind speed at the track and its supporting layer was low (approximately 1 m·s^−1^). With uniform openings, the airflow diffused after passing through the windshield wall due to the existence of openings, and the velocity distribution in the vortex zone was uneven, while the wind speed at the track and its supporting layer was approximately 6 m·s^−1^. With a bottom opening, the vortex zone appeared next to the windshield wall, while the track supporting layer at the step became a conflux acceleration zone, and a low velocity vortex zone near the track line^10^,^11^.

**Figure 5:**
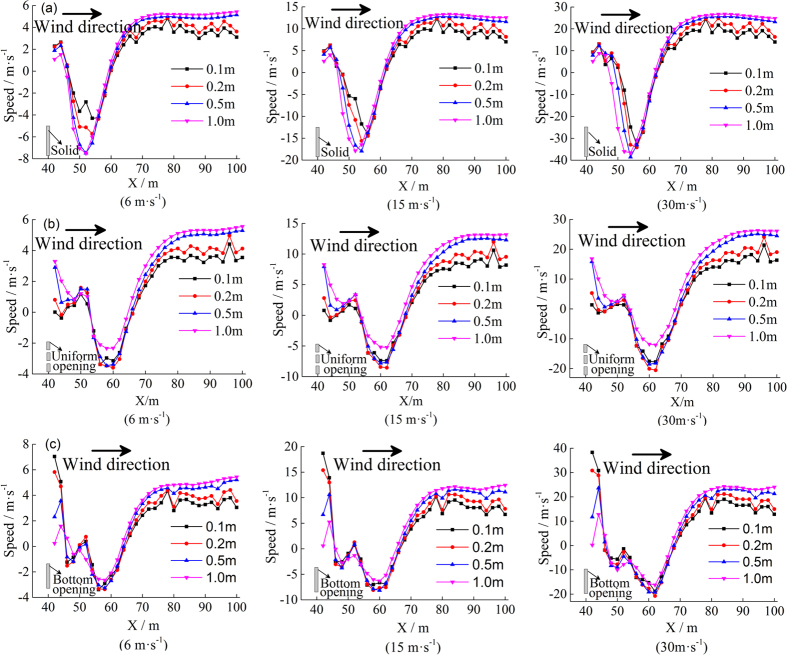
Change of wind speed at different distances from the surface for different types of windshield walls. (**a**) Solid type, (**b**) uniform-opening type, and (**c**) bottom-opening type.

To study the change of the flow field under the influence of three types of windshield walls, the wind speeds at different distances of the leeward side of windshield walls were extracted to analyze resulting changes of wind speeds. The wind speed changes along the horizontal direction at four characteristic heights of 0.1 m, 0.2 m, 0.5 m, and 1.0 m on the leeward sides of three types of windshield walls are shown in Fig. 5. Figure 5 reveals that the velocity of airflow between the solid windshield wall and the track first increased and then decreased, while the velocity of airflow on the leeward side of the track line decreased to a minimum. The airflow velocity on the leeward side of the uniform-opening windshield wall first decreased and then increased, and when the inlet wind speed was 6 m·s^−1^, the airflow speed at both up and down the track lines and the windward side remained below 5 m·s^−1^ (wind speed to blow the sand). This result indicates that at this time, there was substantial sand sediment at the windward side and up-down lines of the track. With increasing inlet wind speed, the velocity of up-down lines and at the windward side of the track increased (in negative direction), and the distribution of sand sediment gradually moved backward. Comparing the airflow change behind the first two types of windshield walls reveals that the attenuation of the airflow velocity at different heights of the bottom-opening windshield wall differed. Within the influence range of the opening, the attenuation of airflow was approximately 82.37% at an inlet wind speed of 6 m·s^−1^, reaching more than 80% at inlet wind speeds of 15 m·s^−1^ and 30 m·s^−1^. However, analyzing the wind velocity near the track revealed that for an inlet wind speed of 6 m·s^−1^ (which was below the wind speed to blow the sand at up-down lines of the track), the majority of sand remained on the track. When inlet speeds were 15 m·s^−1^ and 30 m·s^−1^, the velocity of sand-carrying airflow at up-down lines of the track gradually increased, and the sand sediment area decreased in size, gradually moving backward.

### Analysis of sand sediment

**Figure 6:**
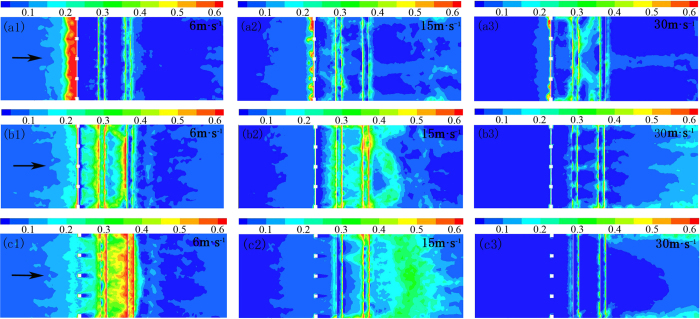
Distribution of sand sediment along the track for three windshield walls under different wind speeds (**a**) Solid type, (**b**) uniform-opening type, and (**c**) bottom-opening type. (Different colors represent the distribution of sand sediment, where the red color indicates the largest amount of sand sediment, while the blue color indicates no sand sediment and other colors indicate the movement of sand) Note: the number on the ruler indicates sand volume percentages (in %).

Wind is the power condition that starts sand movement. For wind speeds above the threshold wind speed of sand, the sand begins to jump and move with the wind. When the wind speed is affected by obstacles and is below those starting the sand, the sand will settle around the obstacles and form sand sediment. The porosity characteristics of the windshield wall affect the movement direction and volume of both front and back airflows, thus determining sediment morphology. To control the sand along the railway, the windshield wall should let the sand sediment settle on the windward side or close to the leeward side of the windshield wall to avoid burying the railway embankment and rail tracks in sand. Figure 6 depicts the distributions of sand sediment around the windshield wall and on the track line for three wind speeds. Figure 6 reveals that for an incoming wind speed of 6 m·s^−1^, the sand under the influence of the solid windshield wall was mainly distributed on the windward side, while only a small amount of sediment was concentrated on the leeward side. This result was in line with the sand control project requirements. With uniform and low openings, the sand mainly distributed on the leeward side, mostly located along the track.

**Figure 7:**
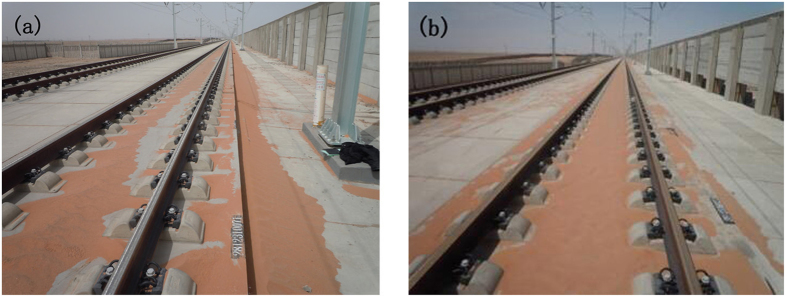
Realistic observations of sand sediment along the track (**a**) Solid type and (**b**) bottom-opening type.

The windward side accumulated only a small amount of sediment; however, the openings enabled the sand to enter that was originally blocked outside of the windshield wall, indicating a poor sand removal effect. For wind speeds of 15 m·s^−1^ and 30 m·s^−1^, sand sediment in the windward side of the solid windshield wall gradually decreased, while the accumulated sand increased in the leeward side and the track line. Under the influence of the uniform-opening windshield wall, the up-down track lines gradually decreased and moved backward. However, the effect was not strong. Analysis of the distribution of sand sediment under the influence of a bottom-opening windshield wall revealed that the sand sediment on up-down track lines significantly decreased, which represented an obvious backward shift. However, since the track structure was still a small sand-blocking structure, a small amount of sediment remained at the inner side of the track. However, the total volume of sand sediment decreased significantly. Figure 7 shows the distribution of sand sediment monitored along the railway in the “Yan Dun” wind zone. Figure 7 reveals that the sand sediment mainly concentrated on the uplink track and its supporting layer under the effect of a solid windshield wall, while almost no sand was found on the downlink and the track line. In case of bottom openings, after a certain period, the majority of sand sediment was concentrated on the track line under the influence of the windshield wall (with low openings). Furthermore, there was some sand sediment between the uplink and downlink, indicating a backward shift of the sand sediment area.

The above analysis suggests that the sand control effect of the windshield walls relates to the form of the windshield walls and the incoming wind speed. If we want to achieve a satisfying sand control effect, the actual project should take full account of the form of the windshield walls and local wind speed conditions. In areas with low wind speeds, solid windshield walls are sufficient and should be installed, since they achieve good sand controlling effect and facilitate convenient construction. However, in areas with high wind speeds, bottom-opening windshield walls should be installed to achieve a good sand control effect.

### Wind tunnel experimental results

#### Change law of flow field

**Figure 8:**
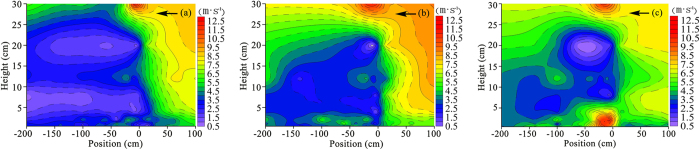
Contours of wind velocity for different windshield walls (**a**) Solid type, (**b**) uniform-opening type, and (**c**) bottom-opening type.

The flow field data of the wind tunnel experiment were measured via pitot tube. The measured data were processed via Excel and kriging interpolated via Sufer11.0 to draw a wind speed contour map. Figure 8 shows the contours of wind velocity at a wind speed of v = 15 m·s^−1^ for three windshield wall models. The distribution of experimental flow field in the wind tunnel reveals that the effect of three types of windshield walls in the vortex zone was very different: the vortex zone area on the leeward-side of a solid windshield wall was relatively large, and the velocity of sand-carrying airflow ranged between 0 and 2.5 m·s^−1^, while a slight, yet abrupt change in the vortex zone near the surface was observed. The uniform-opening windshield wall had a large amount of air permeation near the surface, and the leeward side was composed of multiple vortex flows at places of abrupt change. Due to a further increase in air permeation near the surface at the bottom-opening windshield wall, the characteristics of the vortex zone were completely disrupted. The wind velocity near the leeward-side of the windshield wall was larger than the wind speed blowing the sand, and then gradually decreased to below where wind speed will blow the sand. This indicates the existence of a minor sand sediment near the leeward side of the windshield wall, which was concentrated at some distance from the leeward side of the windshield wall. Comparing this with the numerical simulation of flow field characteristics (Fig. 4) reveals that the wind tunnel experiment is in good agreement with the numerical simulation of change in the flow field, thus verifying the accuracy of these simulation results.

### Analysis of sand sediment

**Figure 9:**
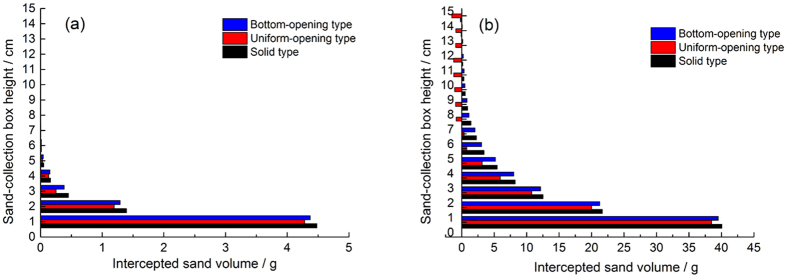
Change of intercepted sand volume for different types of windshield walls (**a**) 6 m·s^−1^ and (**b**) 15 m·s^−1^.

**Figure 10:**
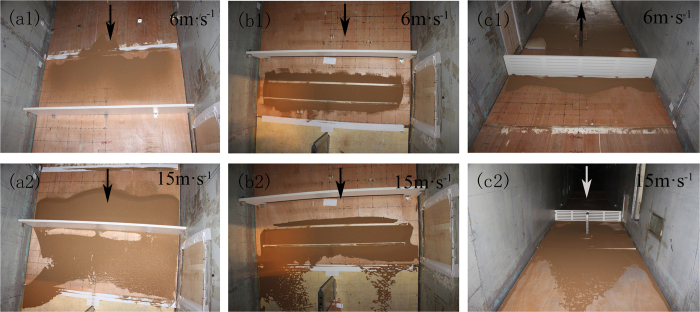
Distribution of sand sediment for different windshield walls (**a**) Solid type, (**b**) bottom-opening type, and (**c**) uniform-opening type.

Sand movement in the vertical axis exhibits layered characteristics. When sand-enriched airflow encounters a sand barrier, changes in the sand-flow field result in different quantities of sand passing through at different heights. Figure 9 shows a plot of intercepted sand volume for three windshield wall models after about 1 min of continuous wind, blowing at speeds of 6 m·s^−1^ and 15 m·s^−1^. The results reveal that with the increase of sand-collection box height, the intercepted sand amount of all three types of windshield walls decreased, indicating that the intercepted sand amount of the windshield wall decreases with the increase of height, and this change is severe for low height. For a wind speed of 6 m·s^−1^, the intercepted sand volume of the solid windshield wall is maximal, while that of the bottom-opening windshield wall is minimal. This indicates that the sand sediment volume in the windward side of the solid windshield wall is higher than that of the bottom-opening windshield wall. However, when the wind speed is 15 m·s^−1^, the changing trend of the intercepted sand amount of the solid windshield wall is similar to that of the bottom-opening windshield wall. This is an indication that the distributions of sand sediment are approximately equal at location 3 H of the leeward-side of the windshield wall. This result also shows that the intercepted sand volume of the uniform-opening windshield wall is minimal and even exhibits negative values, indicating a lower capacity to intercept sand. The reason is that the uniform-opening windshield wall has large wind permeation and enhanced capacity to carry sand, thus increasing the amount of sand sediment in the sand collection box. Figure 10 shows the distributions of sand sediment in the leeward side of three windshield walls (wind direction is depicted with arrows). Comparing the numerical simulation and *in situ* measurements reveals that the distributions of sand sediment in the wind tunnel experiment and numerical simulation (Fig. 6) are in good agreement.

## Discussion

Analysis of these results reveals that a solid windshield wall can achieve a good sand control effect for low incoming wind velocities, and the bottom-opening windshield wall improves this sand control effect for high wind speeds. The sand removal effect of the uniform-opening windshield wall lies between both of these. Considering hydrodynamic mechanisms, the flow field structure on the leeward side of different types of windshield structures is a result of convergence and diffusion of fluids caused by an obstacle. This convergence and diffusion effect of air fluid is more apparent at high wind velocities, but not obvious at low wind velocities.

**Figure 11:**
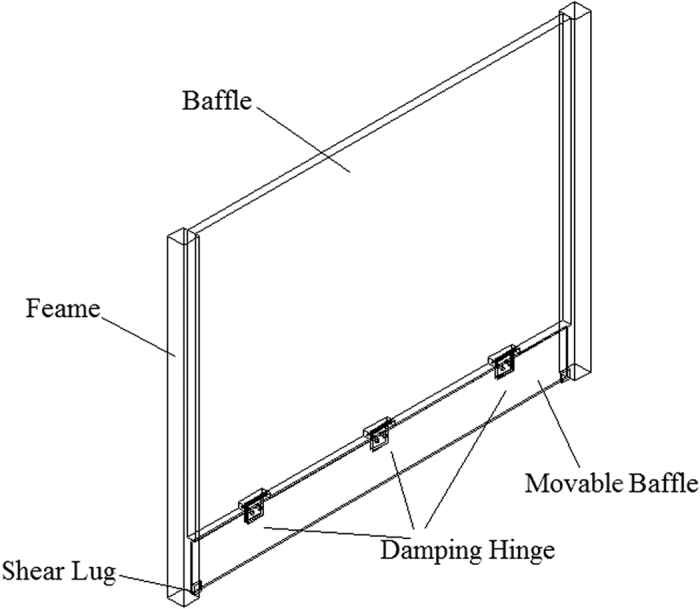
Structure of the window-type windshield wall.

Based on the statistics of wind speed in the “Yan Dun” wind zone, the maximal annual wind speed is approximately 25 m·s^−1^, while the average wind speed is approximately 12 m·s^−1^. Considering the wind-proof effect under different wind speed conditions throughout the year, the above three types of windshield walls cannot achieve satisfying results. Combing the sand control effect at different incoming wind speeds of three kinds of windshield walls, bottom-opening and solid windshield walls were optimized and modified. Consequently, a novel window-type windshield wall was proposed considering both characteristics. The window-type windshield wall adopts a movable baffle on the basis of the bottom-opening windshield wall. The movable baffle is connected to the fixed baffle via damping hinges. Shear lugs are installed on the windward side of the frame columns so that the fixed baffle cannot move to the windward side. The structure is shown in Fig. 11. The movable baffle in the window-type windshield wall can only be opened in a fixed direction, and will only open when the wind speed is above a critical value. For low incoming wind speeds, the movable baffle does not open due to the self-weight of the movable baffle and the effect of damping hinges. Therefore, airflow is prevented from depositing sand to the windward side near the track in the leeward-side via compression acceleration. Such an effect is equivalent to the sand control mechanism of the solid windshield wall. For high incoming wind speeds, the movable baffle will overcome its self-weight and the damping force will open towards the leeward-side. Due to high incoming wind speeds, the sand in the vicinity of the windshield wall can be blown far away subsequent to the compression acceleration of the baffle to avoid sand sediment accumulation in the track lines. Such an effect is equivalent to the sand control mechanism of the bottom-opening windshield wall. The advantage of the window-type windshield wall is the design of movable baffles that have the ability to open and close. Combined with the effect of damping hinges, such a design allows the movable baffle to open in one direction only. Therefore, when the wind speed exceeds a critical value, the baffle will automatically open and thus, effectively reduce the sand sediment at low wind speeds. Consequently, the windshield wall can achieve better sand-proof effect with high practical value for a variety of wind speeds.

The relationship between the movable baffle, the incoming wind speeds, and the specific design of the baffle in the window-type windshield wall will be analyzed in detail in a follow-up article. The purpose of this study was to clarify the sand-proof benefit of different types of windshield walls along the railway within the “Yan Dun” wind zone, summarizing the rules to propose an ideal window-type windshield wall.

Our results show that (1) One year of wind speed monitoring in the “Yan Dun” wind zone revealed average annual wind speeds of approximately 12 m·s^−1^. The maximum observed wind speed was 25.3 m·s^−1^ and the minimum wind speed was 3.4 m·s^−1^; (2) Results obtained from the wind tunnel experiment and numerical simulation revealed different flow field characteristics on the leeward-side of three types of windshield walls. Apparent vortex and acceleration zones were formed in the leeward-side of the solid windshield wall, leaving the track and its supporting layer exposed to wind speeds below speeds that blow the sand. Due to uniform openings, the airflow exhibits diffusion after passing through the windshield wall, and the track and its supporting layer are exposed to wind speeds above speeds that blow the sand. In case of bottom openings, the vortex zone appears close to the windshield wall. Furthermore, the track supporting layer at the step becomes a conflux acceleration zone, while the vortex zone with low speed is near the track line; (3) Two types of test results show that at different incoming wind speeds, three types of windshield walls have different sand controlling effects. At a relatively low wind speed (6 m·s^−1^), the solid windshield wall achieves a good sand controlling effect, while the openings introduce sand that was originally blocked by the windshield wall. When the wind speed increases, the bottom-opening windshield wall achieves better sand removal effect. However, since the track structure still represents a small sand-block structure, a small amount of sand sediment still enters the track. However, the total volume of the sand sediment decreases significantly. The sand control effect of the uniform-opening windshield wall lies between the different types; (4) After clarifying the sand control effects of three types of windshield walls at different wind speeds, a novel type of windshield wall was proposed, taking different wind speeds into account: the window-type windshield wall. Using a movable baffle, the window-type windshield wall can achieve an adequate sand-proofing effect at different wind speeds, thus representing high practical value.

## Methods

### Field observations

The field observation site is located in the “Yan Dun” wind zone along the Lanxin Railway II, with a mileage starting from DK1228 + 100 and ranging to DK1228 + 200, and a total length of the railway of 100 m. The type of utilized windshield wall was solid. The sand sediment was first observed, then a 1 m high concrete slab was removed from the bottom of the wall and after a month the sand sediment was observed again. The observation time was from April to May (where average wind speed was approximately 14 m·s^−1^, while the maximum wind speed was approximately 26 m·s^−1^). The method of observation was mainly via *in situ* artificial image acquisition, and qualitative analysis was carried out on the sand sediment characteristics along the test section railway track and the nearby support layer.

### Numerical model

#### Geometrical model

**Figure 12:**
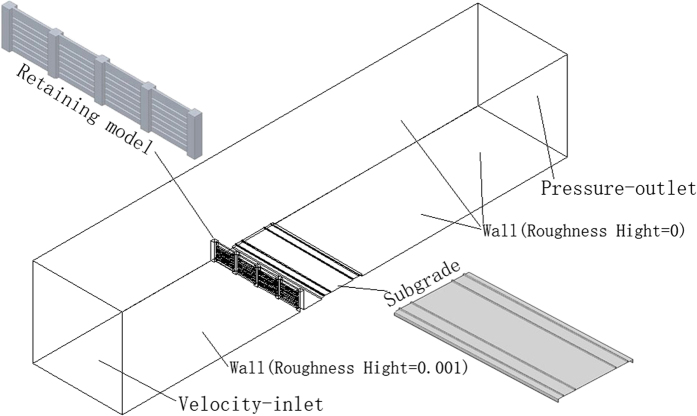
Windshield wall model and utilized boundary conditions.

A three-dimensional model was established in AUTOCAD, meshed, and numerically simulated in CFD software, and then post-processed in TECPLOT. The calculation area was 20 m high, 20 m wide, and 100 m long. As shown in Fig. 12, the windshield wall was 3.8 m high and 0.3 m thick. The model inlet condition was VELOCITY-INLET and the outlet condition was PRESSURE-OUTLET with a differential pressure of zero.

#### Mesh generation

Due to the existence of the windshield wall, the resulting calculation area of the model was irregular. Therefore, the tetrahedron mesh was adopted for meshing the model, and the patch dependent method was adopted for partitioning. To better capture the flow field and sand sediment changes near the surface and in the vicinity of the windshield wall, inflation encryption was used around the windshield wall. The first layer height was designated Y + , while the number of encryption mesh layers was 20 with a rate of increase of 1.1. The total number of mesh in the calculation area was approximately 6 million.

#### Calculation parameters

The particle size of the sand conveyed by the sandy air is typically 0.075–0.25 mm. In this study, the particle size of the sand (d_s_) was 0.15 mm, the density of the sand (ρs) was 2650 kg·m^−3^, and the viscosity (μ) was 0.047 Pa·s^12^. The initial sand volume fraction was 1.5%, air density (ρ) was 1.225 kg·m^−3^, and the viscosity (μ) was 1.789 × 10^−5^ Pa·s^13^. The pressure was ordinary and the inlet was a typical wind profile flow, i.e.,


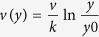


where v is the friction wind speed; y_0_ is the rough length; k is von Karman coefficient (used as 0.4); y is height; and *v(y*) is the wind speed value at the height of y.

#### Governing equations and simulation process

According to aerodynamic theory, for a Mach number below 0.3, the flow is incompressible. In this study, the Mach number of the sand flow was below 0.3; therefore, it could be regarded as incompressible flow^14^,^15^,^16^. The governing equations for the simulation of incompressible fluids include the continuous equation, the momentum equation, and the k-ε turbulence model equation. Detailed equations have been presented in a previous publication^17^. To retain the same principles as in the wind tunnel test, the numerical simulation first carried out the net wind experiment, followed by the blowing sand experiment.

### Wind tunnel experiment

**Figure 13:**
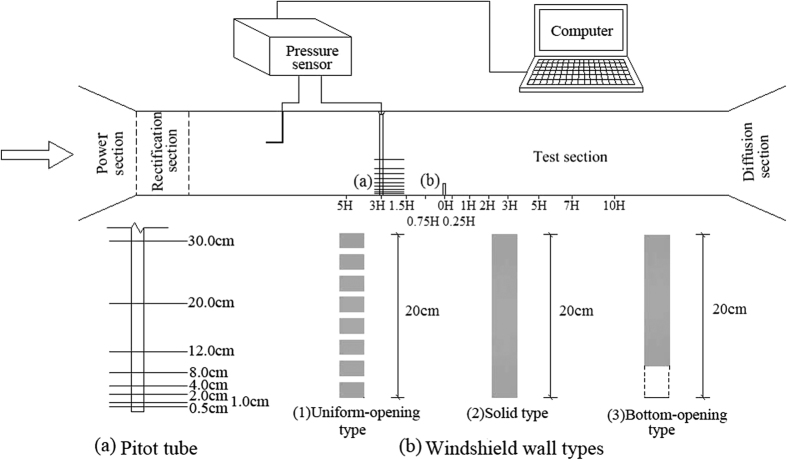
Test principle of wind tunnel experiment and design of test section.

The wind tunnel experiment was carried out in the wind tunnel laboratory of the Cold and Arid Regions Environmental and Engineering Research Institute of the Chinese Academy of Sciences. This wind tunnel belongs to direct-current blowing environmental wind tunnels and consists of five parts: power section, rectification section, sand supply device, test section, and diffusion section. The length of the wind tunnel was 38.0 m, while the length of the test section was 21.0 m with a rectangular cross section of 1.2 m × 1.2 m. The layout of the experimental model in the wind tunnel is shown in Fig. 13 and the height of the model was 20 cm.

Since the wind tunnel has a significant influence on the leeward-side flow field, more monitoring points of the wind tunnel test were introduced on the leeward side than on the windward side^18^.

Monitoring points were installed in the location of the windshield wall, at 0 H and the model windward-side locations 0.75 H, 1.5 H, 3.0 H, and 5.0 H, as well as at leeward-side locations −0.25 H, −0.5 H, −1.0 H, −2.0 H, −3.0 H, −5.0 H, −7.0 H, and −10.0 H, respectively. The measured data were processed to form a flow field distribution map. Wind speed measuring points were set up at 0.5 cm, 1.0 cm, 2.0 cm, 4.0 cm, 8.0 cm, 12.0 cm, 20.0 cm, and 30.0 cm at the vertical height of the Pitot tube. The inlet wind speeds were set at 6 m·s^−1^, 9 m·s^−1^, 12 m·s^−1^, and 15 m·s^−1^, respectively.

The flow field test was first carried out on three types of windshield wall models. The sand source was then placed and the sand was continuously blown for approximately 1 min. The sand sediment area and sediment volume were observed in front of and behind of each model, and a sand collection box was set up at location 3 H, leeward of the windshield wall. The sand sediment volume was measured under three types of opening distributions. The effect of windshield walls on the distribution of sand sediment was studied via comparison with sand sediment volumes in the sand collection box in the open field (with identical wind speed and sand source, but without windshield walls).

## Additional Information

**How to cite this article**: Cheng, J.-J. *et al*. Unloading Characteristics of Sand-drift in Wind-shallow Areas along Railway and the Effect of Sand Removal by Force of Wind. *Sci. Rep.* **7**, 41462; doi: 10.1038/srep41462 (2017).

**Publisher's note:** Springer Nature remains neutral with regard to jurisdictional claims in published maps and institutional affiliations.

## Figures and Tables

**Figure 1:**
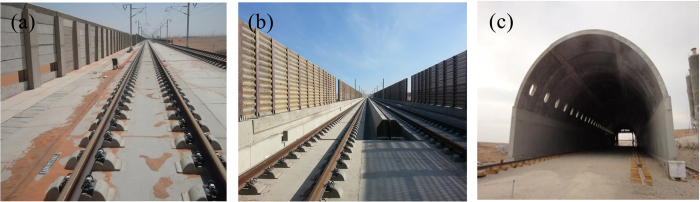
Main windshield engineering structures along the Lanxin Railway II. (**a**) Subgrade windshield walls, (**b**) bridge windscreens, and (**c**) wind-proof tunnels.

## References

[b1] Zhang J. P., Wang Y. S. & Jiang F. Q. Numerical analysis on the features of sand flow movement around the embankment of Lan-Xin railway in Gobi region. China Railway Science. 32. 14–18 (2011).

[b2] Cheng J. J.. Characteristics of thedisastrouswind-sand environment along railways in the Gobi area of Xinjiang, China. Atmospheric Environment. 102, 344–354 (2015).

[b3] Cheng J. J., Lei J. Q., Li S. Y. & Wang H. F. Disturbance of the inclined inserting-type sand fence to wind-sand flow fields and its sand control characteristics. Aeolian Research. 21, 139–150 (2016).

[b4] Zhang K. C., Qu J. J., Han Q. J. & An Z. S. Wind energy environments and aeolian sand characteristics along the Qinghai-Tibet railway, China. Sedimentary Geology. 273, 91–96 (2012).

[b5] Ellis J. T. & Sherman D. J. Fundamentals of aeolian sediment transport: wind-blown sand. In: shroder J. F. (Ed.), treatise on geomorphology. Academic Press, San Diego, pp. 85–108 (2013).

[b6] Grafals-Soto R. & Nordstrom K. Sand fences in the coastal zone: intended and unintended effects. Environmental Management. 44, 420–429 (2009). 19629579 10.1007/s00267-009-9331-7

[b7] Xu X. L., Zhang K. L., Kong Y. P., Chen J. D. & Yu B. F. Effectiveness of erosioncontrol measures along the Qinghai-Tibet highway, Tibet plateau, China. Transport Res D: Tr E. 11, 302–309 (2006).

[b8] Zhang K. C., Qu J. J., Liao K. T., Niu Q. H. & Han Q. J. Damage by windblown sand andits control along Qinghai-Tibet railway in China. Aeolian Research. 2, 143–146 (2010).

[b9] Huang N. & Zheng X. J. Theoretical simulation of developing process of wind-blown sand movement. Key Engineering Materials. 243-244. 589–594 (2003).

[b10] Jiang H., Huang N. & Zhu Y. J. Analysis of wind-blown sand movement over transverse dunes. Scientific Reports. 4, doi: 10. 1038 / srep 07114 (2014). 10.1038/srep07114 PMC4248282 25434372

[b11] Parsons D. R., Wiggs G. F., Walker I. J., Ferguson R. I. & Garvey B. G. Numerical modelling of airflow over an idealised transverse dune. Environmental Modelling & Software. 19, 153–162 (2004).

[b12] Huang N., Zheng X. J. & Zhou Y. H. A multi-objective optimization method for probability density function of lift-off speed of wind-blown sand movement. Advances in Engineering Software. 37, 32–40 (2006).

[b13] Bean A., Alperi R. W. & Federer C. A. A method for categorizing shelterbelt porosity. Agricultural and Forest Meteorology. 14, 417–429 (1975).

[b14] Taniere A., Oesterle B. & Monnier J. C. On the behaviour of solid particles in a horizontal boundary layer with turbulence and saltation effects. Experiments in Fluids. 23, 463–471 (1997).

[b15] Li B. J. & Douglas J. Sherman. Aerodynamics and morphodynamics of sand fences: a review. Aeolian Research. 17, 33–48 (2015).

[b16] Cheng J. J., Jiang F. Q., Yang Y. H. & Xue C. X. Study on the hazard characteristics of the drifting sand along the railway in Gobi area and the efficacy of the control engineering measures. China Railway Science. 31, 15–20 (2010).

[b17] Lee S. J., Park K. C. & Park C. W. Wind tunnel observations about the shelter effect of porous fences on the sand particle movements. Atmospheric Environment. 36. 1453–1463 (2002).

[b18] Frank A. J. & Kocurek G. Toward a model for airflow on the lee side of aeolian dunes. Sedimentology. 43. 451–458 (1996).

